# Analysis of Risk Factors for Kidney Retransplant Outcomes Associated with Common Induction Regimens: A Study of over Twelve-Thousand Cases in the United States

**DOI:** 10.1155/2017/8132672

**Published:** 2017-09-24

**Authors:** Alfonso H. Santos, Michael J. Casey, Karl L. Womer

**Affiliations:** Department of Medicine, Division of Nephrology, Hypertension and Renal Transplantation, University of Florida, Gainesville, FL, USA

## Abstract

We studied registry data of 12,944 adult kidney retransplant recipients categorized by induction regimen received into antithymocyte globulin (ATG) (*N* = 9120), alemtuzumab (*N* = 1687), and basiliximab (*N* = 2137) cohorts. We analyzed risk factors for 1-year acute rejection (AR) and 5-year death-censored graft loss (DCGL) and patient death. Compared with the reference, basiliximab: (1) one-year AR risk was lower with ATG in retransplant recipients of expanded criteria deceased-donor kidneys (HR = 0.56, 95% CI = 0.35–0.91 and HR = 0.54, 95% CI = 0.27–1.08, resp.), while AR risk was lower with alemtuzumab in retransplant recipients with >3 HLA mismatches before transplant (HR = 0.63, 95% CI = 0.44–0.93 and HR = 0.81, 95% CI = 0.63–1.06, resp.); (2) five-year DCGL risk was lower with alemtuzumab, not ATG, in retransplant recipients of African American race (HR = 0.54, 95% CI = 0.34–0.86 and HR = 0.73, 95% CI = 0.51–1.04, resp.) or with pretransplant glomerulonephritis (HR = 0.65, 95% CI = 0.43–0.98 and HR = 0.82, 95% CI = 0.60–1.12, resp.). Therefore, specific risk factor-induction regimen combinations may predict outcomes and this information may help in individualizing induction in retransplant recipients.

## 1. Introduction

Based on the United States Renal Data System (USRDS) report in 2013, 14.3% of patients in the renal transplant waitlist were retransplant candidates and 11.5% of kidney allografts went to recipients with previous kidney transplants [1]. Retransplant recipients have survival rates that are superior to waitlisted patients with failed allograft and comparable to primary transplant recipients; but, the survival of retransplants are inferior to that of primary allografts [2–8].

The effects of induction agents on the outcome of kidney transplants have been more extensively investigated in primary than in repeat transplants. Studies have shown that basiliximab reduces acute rejection rates better than placebo and has a comparable effect on acute rejection rates as antithymocyte globulin (ATG) or alemtuzumab in low risk patients; but, ATG and alemtuzumab are more efficacious in reducing acute rejection rates in high risk patients [9–16]. Since recipients of kidney retransplants are at high immunological risk for rejection and complications of immunosuppression, for this group of patients, a careful and thoughtful induction selection is crucial to achieve succesful outcomes [17]. A recent large registry analysis showed that in kidney retransplants, short-term outcomes such as delayed graft function, acute rejection, BK virus infection and patient mortality did not differ between induction groups; although, alemtuzumab was associated with a higher risk of graft-failure [18]. In the rest of our literature review, we encountered studies that identified factors affecting outcomes in primary and repeat kidney transplants, but we have not seen an analysis on the impact of the interaction between risk factors and induction regimens on patient and allograft outcomes after kidney retransplant [1, 16, 19–27]. Thus, there is a dearth of information to guide practitioners in utilizing practical clinical data in the selection of induction regimens for kidney retransplant recipients. Therefore, we conducted this retrospective analysis of kidney retransplant outcomes in 12,944 adult recipients based on a 14-year data of the SRTR. Using time-survival models, we determined the 1-year rejection-free graft survival rates and 5-year patient and death-censored graft survival rates of retransplant recipient cohorts given ATG, alemtuzumab, or basiliximab induction. Multivariable adjusted analyses showed that the significance and strength of associations between retransplant outcomes and the combinations of risk factor and induction regimen were not always uniform and, in fact varied in few instances. This report identifies risk factor and induction regimen combinations with different relative risks for one-year acute rejection and five-year graft loss and patient death after kidney retransplant. Our findings would contribute towards the individualized selection of induction regimen for kidney retransplants based on risk factors assessment.

## 2. Patients and Methods

### 2.1. Data Source

The University of Florida Institutional review board approved this study which used data from the Scientific Registry of Transplant Recipients (SRTR). The SRTR system includes data on all donor, waitlisted candidates, and transplant recipients in the US, submitted by the members of the Organ Procurement and Transplantation Network (OPTN), and has been described elsewhere [28]. The Health Resources and Services Administration provides oversight to the activities of the OPTN and SRTR contractors.

### 2.2. Study Design and Population

This is an observational retrospective cohort study based on the data from the Scientific Registry of Transplant Recipients (SRTR) that included patients aged 18 years old and above with previous kidney transplant(s) and received a repeat kidney transplant (also termed retransplant) between Jan. 1, 2003 and Dec. 31, 2013. Only repeat kidney transplant recipients [also termed retransplant recipients] who received anti-thymocyte globulin (ATG), alemtuzumab, and basiliximab for induction immunosuppression and tacrolimus with mycophenolate (with or without steroids) for maintenance immunosuppression were included in this study.

Retransplant recipients were categorized into three cohorts based on receipt of one of the above induction regimens at the time of retransplant surgery and were excluded if they did not receive an induction or had received other induction agents, had other organ transplant/s, had received maintenance immunosuppression other than the tacrolimus and mycophenolate (with/without steroids) regimen, or had a missing pretransplant panel reactive antibody (PRA) result in SRTR.

Study entry was defined as the date of kidney retransplant between January 1, 2003 and December 31, 2013, and follow-up was terminated at the earliest of (1) end of the 5-year observation, (2) end of SRTR follow-up, (3) loss to follow-up (4) subsequent kidney retransplantation, or (5) death. Study outcomes included patient survival and risk factors for death at 5-year, overall, and death-censored graft survival and risk factors for death-censored graft loss at 5-year and acute rejection-free survival and risk factors for acute rejection at 1 year. Overall graft loss was defined as return to dialysis, retransplantation, or death, while death-censored graft loss was defined with the first two of the preceding criteria. Acute rejection was defined as biopsy-proven rejection or treated rejection censored for graft loss or death based on the SRTR standard analysis file. To minimize the confounding effect of variations in maintenance immunosuppression regimens on the transplant outcomes, we restricted our analysis to retransplant recipients on a maintenance regimen containing only tacrolimus and mycophenolate* with or without* steroids at the time of discharge from the index retransplant surgery. The tacrolimus and mycophenolate regimen was chosen as the standard maintenance immunosuppression based on SRTR data showing that it has been the predominant regimen in around 92% of kidney transplants in the USA [29]. Steroids maintenance regimen was controlled for in the multivariable models. Collected data included demographic and medical information of transplant donors and recipients as well as clinical factors pertinent to the transplant operation (Table 1).

### 2.3. Statistical Analysis

Categorical data were presented as frequencies and percentages and compared using Chi-square test. Continuous variables were presented as means and standard deviations and compared using* F* or Student's *t*-test. Kaplan-Meier curves with log-rank testing were used to analyze patient survival rates; acute rejection-free graft survival rates; and overall and death-censored graft survival rates of the induction cohorts studied. Multivariable Cox proportional hazards models (also termed Cox models) were used to assess the role of induction agents as independent risk factors for the outcomes. Cox models were also used to assess the interaction effect of induction agents and risk factors on outcomes. Covariates used in the Cox models included clinically relevant risk factors from Table 1. An additional “missing variable category” was created for any covariate with incomplete data [30]. No data was imputed. Conformity of the models with the Cox proportional hazards assumption was verified by visual inspection of Schoenfeld residual plots for the explanatory variables fitted in the model [30, 31]. For this study, all analyses were performed using SAS software, version 9.4 (SAS Institute, Inc., Cary, NC, USA). Statistical significance was identified by a* p *value of ≤ .05, and all confidence intervals used a 95% threshold.

## 3. Results

### 3.1. Study Population and Demographics

After screening, we studied 12,944 eligible adults receiving repeat kidney transplant/s between January 1, 2003, and December 31, 2013. Among these, 9120 (70.5%) received antithymocyte globulin (ATG), 1687 (13.0%) received alemtuzumab, and 2137 (16.5%) received basiliximab for induction. Maintenance immunosuppression included corticosteroids in 84% of the ATG, 52% of the alemtuzumab, and 89% of the basiliximab induction cohorts (*p* < .001). The baseline characteristics of the study cohorts are shown in Table 1. The mean patient age in the cohorts ranged between 44 and 46 years old. Compared with the basiliximab cohort, the ATG and alemtuzumab cohorts had higher proportions of African American recipients (14.7% versus 22.8% and 23.1%, resp.; *p* < .001) and donors (8.9% versus 12.6% and 12.2%, resp.; *p* < .001). The basiliximab cohort had the highest percentage of living donor kidney retransplant recipients (43.4%) compared with the other two cohorts (ATG = 28.9% and alemtuzumab = 34.1%) (overall *p* < .001).

### 3.2. Presentation of Outcomes Analyses

For each outcome, analyses adjusted for inductions and risk factors main effects (without interaction terms) are displayed in Figure 2; and comparison of interactions between risk factor and inductions is displayed in Figures 3, 4, and 6; respectively.

### 3.3. Patient Survival and Risks for Death

**Figure 1:**
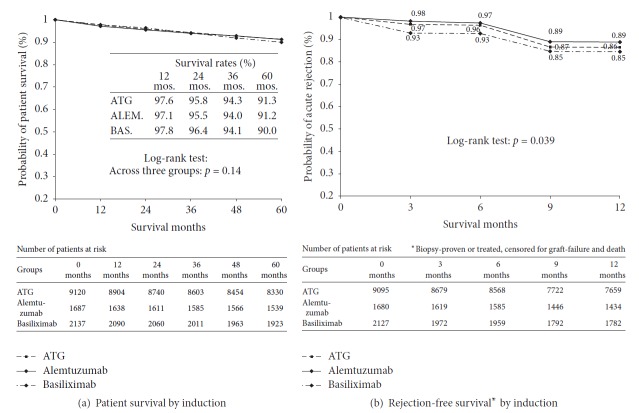


Based on time-survival curves (Figure 1(a)), the one-year and five-year survival rates of patients in the three induction cohorts were 97.6% and 91.3% for ATG, 97.1% and 91.2% for alemtuzumab, and 97.8% and 90% for basiliximab, respectively (log-rank *p* = .14).

**Figure 2:**
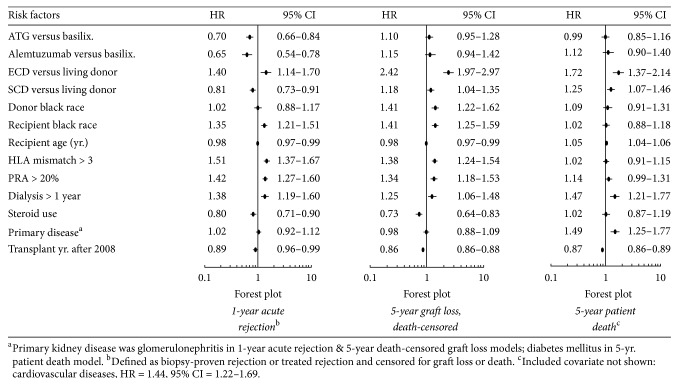
Kidney retransplantation outcomes: main Cox model (no interactions).

**Figure 3:**
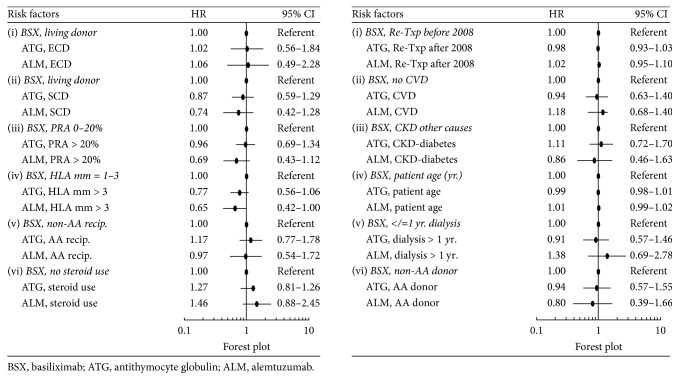
Patient death in 5 years of kidney retransplant, Cox model with interactions.

Significant risk factors for patient death in the 5 years following retransplant in the main Cox model (without interaction terms) included ECD or SCD (versus living donor) kidney, older recipient age, >1-year dialysis duration before transplant, cardiovascular disease, and diabetes mellitus (Figure 2). Based on the Cox model with interaction terms, in the presence of >3 recipient-donor HLA mismatches, alemtuzumab, not ATG, seemed to be associated with a lower relative risk of patient death compared with control [(HR = 0.65, 95% CI = 0.42–1.00) and (HR = 0.77, 95% CI = 0.56–1.06), resp.] (Figure 3).

### 3.4. One-Year Acute Rejection-Free Survival and Risks for Acute Rejection

The unadjusted one-year acute rejection-free graft survival rates were 89% for alemtuzumab, 86% for ATG, and 85% for basiliximab (*p* = .039) (Figure 1(b)).

**Figure 4:**
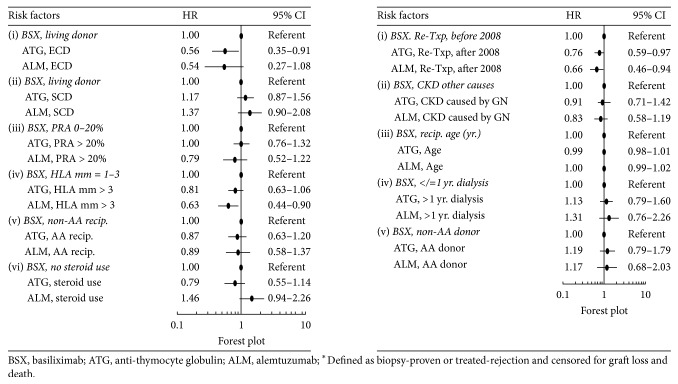
Acute rejection^*∗*^ in first year of kidney retransplant, Cox model with interactions.

The adjusted risk for acute rejection (AR) during the first posttransplant year was significantly lower by 30% for ATG (HR = 0.70, 95% CI = 0.66*–*0.84) and 35% for alemtuzumab (HR = 0.65, 95% CI = 0.54*–*0.78), compared with basiliximab. The risks for AR between ATG and alemtuzumab were not significantly different based on the significant overlap (>50%) in the 95% CIs. The rest of the hazard ratios of risk factors for AR in the main Cox model without interactions are shown in Figure 2. Based on the Cox model with interaction terms in Figure 4, in retransplant recipients with an ECD donor, ATG, not alemtuzumab, was associated with a lower risk for AR compared with control [(HR = 0.56, 95% CI = 0.35–0.91) and (HR = 0.54, 95% CI = 0.27–1.08); resp.]; with >3 donor-recipient HLA mismatches, alemtuzumab, not ATG, was associated with a lower risk for AR compared with control [(HR = 0.63, 95% CI = 0.44–0.90) and (HR = 0.81, 95% CI = 0.63–1.06); resp.]; in the later retransplantation era, ATG and alemtuzumab were both associated with a lower risk for AR compared with control [(HR = 0.76, 95% CI = 0.59–0.97) and (HR = 0.66, 95% CI = 0.46–0.94), resp.].

### 3.5. Death-Censored Graft Survival Rates and Risk Factors for Death-Censored Graft Loss

**Figure 5:**
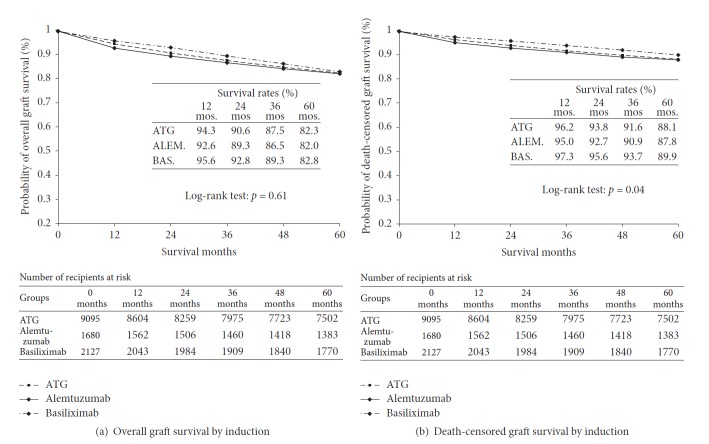


**Figure 6:**
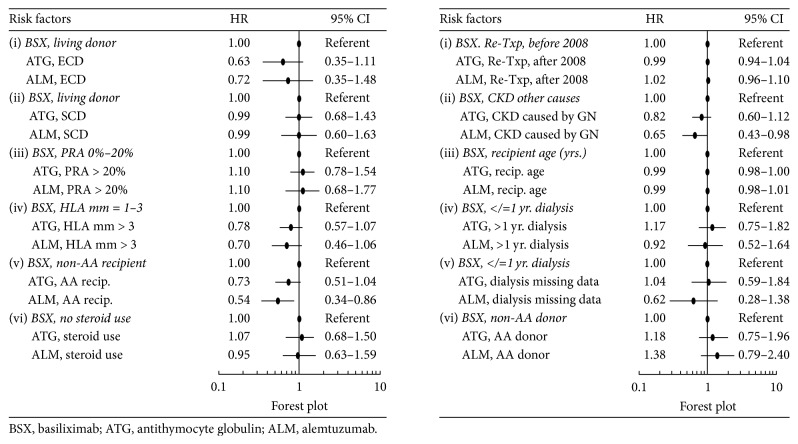
Death-censored retransplant loss in 5 years, Cox model with interactions.

After retransplant, the 5-year overall graft survival rates were 82.3% for ATG, 81.9% for alemtuzumab, and 82.8% for basiliximab (log-rank *p* = .61) (Figure 5(a)). The death-censored graft survival probability rates of the induction cohorts were 88.5% for ATG, 88.2% for alemtuzumab, and 89.9 for basiliximab (log-rank *p* = .04) (Figure 5(b)). Based on the main Cox model, the adjusted risk of death-censored graft loss associated with ATG or alemtuzumab was not different from basiliximab in the five years following retransplant [(HR = 1.10, 95% CI = 0.95–1.28) or (HR = 1.15, 95% CI = 0.94–1.42); resp.] (Figure 2). The rest of the hazard ratios for death-censored graft loss in the main Cox model without interactions are shown in Figure 2. Based on the Cox model with interaction terms in Figure 6, in retransplant recipients of African American race or with primary renal failure due to glomerulonephritis, compared with control, alemtuzumab, not ATG, was associated with a lower risk for 5-year death-censored graft loss [AA race (HR = 0.54, 95% CI = 0.34–0.86, versus HR = 0.73, 95% CI = 0.51–1.04) and GN (HR = 0.65, 95% CI = 0.43–0.98, versus HR = 0.82, 95% CI = 0.60–1.12), resp.].

## 4. Discussion

We retrospectively analyzed SRTR data involving 12,944 repeat kidney transplant (Re-KT) cases from 2003 through 2013 and now present an original report identifying the significant risk factors for acute rejection, graft loss, and death associated with each of the 3 commonly used induction agents in the USA [16]. We found that five-year patient survival rates were not significantly different between the 3 induction cohorts. One-year incidence rates and adjusted risks for AR were lower with ATG or alemtuzumab compared with basiliximab induction. And the five-year adjusted risks for patient death and death-censored graft loss were not significantly different between the three induction agents studied. We identified the risk factor and induction interactions significantly associated with the kidney retransplant outcomes analyzed.

Based on archived SRTR reports, the 1-year patient survival rates for all US adults receiving their primary kidney transplants in 2002-2003 were 94.5%–97.6% and 96%–99% in 2012 [32, 33]. In our analysis, the one-year survival rates of retransplant recipients in the induction cohorts were between 97.2% and 97.9% in 2003–2013 (Figure 1). Our findings are consistent with previous reports that kidney retransplant provides a patient survival rate similar to primary transplant [2–5, 18, 34].

We did not find significant differences in the risks of death associated with the interactions between induction agents and other risk factors shown in Figure 3, except for the suggestively lower risk associated with alemtuzumab with >3 donor-recipient HLA mismatches versus basiliximab with 1–3 HLA donor-recipient HLA mismatches. This relationship was not seen in the comparison between ATG versus control. In a collaborative transplant study report that included 177,584 deceased-donor kidney transplants between 1990 and 2009, Opelz and Döhler found an association between the number of HLA mismatches and risk of death with a functioning graft mainly due to infection and cardiovascular disease [35]. A possible mechanism for the association may have been the need for more intensive immunosuppression as a consequence of increased rejection episodes in transplant recipients with high HLA mismatches [35, 36]. Our results in Figure 4, depicting a significantly lower risk for acute rejection in retransplant recipients with > 3 donor-recipient HLA mismatches given alemtuzumab, may arguably indicate that reduction in AR risk is also the underlying mechanism for the survival benefit of alemtuzumab in retransplant recipients with >3 donor-recipient HLA mismatches in this current study (Figure 3). Our findings need confirmation by future studies as we found no previous evidence that alemtuzumab has reduced the risk of death in primary or repeat kidney transplants with high number of donor-recipient HLA mismatches.

Our analysis showed a lower risk of death-censored graft loss in retransplant recipients with primary diagnosis of GN who received alemtuzumab versus the basiliximab-non-GN control group; this relationship was not seen to be significant with ATG versus the same control group. De novo autoimmune renal conditions such as membranous GN and antiglomerular basement membrane disease have been previously reported in patients receiving alemtuzumab for treatment of multiple sclerosis [37]. A single-center study which included primary and repeat kidney transplants did not show an increased risk of posttransplant GN recurrence in recipients given alemtuzumab compared with interleukin-2 receptor blockers or ATG for induction immunosuppression [38]. Interestingly, in a Korean study, use of basiliximab for induction was found to be a risk factor for posttransplant glomerulonephritis (HR = 1.89, 95% CI = 1.08–1.32) [39]. Despite the larger sample size of the ATG cohort, we did not find a statistically significant HR for death-censored graft loss associated with the ATG × GN compared with the same control (Figure 6). In the context of our study, the results may possibly indicate an undefined difference between the effects of alemtuzumab versus basiliximab in reducing graft loss in retransplant recipients with previous GN.

We found that the adjusted risks of acute rejection in the first year after retransplant were lower with ATG and alemtuzumab compared with basiliximab induction (Figure 3). A systematic review and meta-analysis of ten randomized controlled trials of induction regimens (not exclusive for kidney retransplants) showed similar outcomes of lower rejection risks with ATG and alemtuzumab compared with interleukin-2 receptor antibody induction [9]. We share the opinion of other authors that the high immunological risks of retransplant recipients are mitigated by lymphocyte-depleting, but not by non-lymphocyte-depleting, induction agents such as basiliximab [23, 24, 26, 40, 41].

Consistent with reports favoring the trend of using alemtuzumab induction with steroid-sparing regimens [41, 42], our results in Table 1 showed that the alemtuzumab cohort had the lowest percentage of patients on maintenance corticosteroids after retransplant (52% alemtuzumab, 84% ATG, and 90% basiliximab; *p* < .001), (Table 1). Although steroids use in maintenance immunosuppression was associated with lower risks for acute rejection and death-censored graft loss in the main Cox models (Figure 2), the Cox model with interaction in Figure 6 did not show significant associations between death-censored graft loss and the variable termed: induction agent (ATG and alemtuzumab) × steroid use.

Our main Cox analysis without interaction terms showed that African American (AA) race of a retransplant recipient was a significant risk factor for both retransplant AR and death-censored graft loss (Figure 2). Alemtuzumab induction in AA retransplant recipients was associated with a 46% relative risk reduction for graft loss compared with basiliximab in non-AA retransplant recipients (Figure 6). A 2013 retrospective report by Hussain and colleagues showed that, regardless of induction (alemtuzumab or ATG), graft survival rates did not differ in African American deceased-donor kidney transplant recipients [43]. Another study has shown that alemtuzumab induction eliminates the posttransplant survival disparity between White and African American recipients by improving graft survival in all recipients [44].

In a study of kidney transplants between 2006 and 2014, where the transplant recipients studied were on a uniform maintenance immunosuppression consisting of CNI and mycophenolate, Serrano et al. found that graft outcomes related to alemtuzumab versus ATG induction improved with time due to a “learning curve” effect [42]. In our current analysis, while retransplant in the later era (after 2008) was associated with lower risks of death, AR, and death-censored graft loss in the main Cox models (Figure 2), the interaction models showed that these benefits were not induction agent specific (Figures 3, 4, and 6).

Our study shares the inherent limitations of any retrospective database analysis [45]. We strived to minimize the confounding effect of variation in maintenance immunosuppression regimens on outcomes by limiting the analysis to retransplant recipients on a combined tacrolimus and mycophenolate (with or without steroids) regimen only. Our study has a number of strengths, including the use of a national database where all US transplant centers submitted their transplant-related information. The data we obtained represent real clinical setting experience with a large sample size and follow-up duration unlikely to be achieved in clinical trials.

In summary, we report that, among adult repeat kidney transplant recipients given ATG, alemtuzumab, or basiliximab induction in 2003 to 2013, the 5-year patient and graft survival rates were not independently influenced by the induction agents alone; but one-year posttransplant acute rejection rates were lower with ATG and alemtuzumab compared with basiliximab. The 5-year patient mortality risk seemed to be lower with alemtuzumab induction in retransplants with >3 donor-recipient HLA mismatches compared with the control cohort. Five-year graft loss risk was lower with alemtuzumab induction in recipients with African American race or primary kidney diagnosis of glomerulonephritis compared with the control cohort.

## Figures and Tables

**Table 1:** Demographic and Clinical characteristics of adults receiving repeat kidney transplants in the United States (*N* = 12,944) from 2003 through 2013.

Variables	Antithymocyte globulin	Alemtuzumab	Basiliximab	*p* Value
	*N* = 9120	*N* = 1687	*N* = 2137	
Donor type:				<.001
Expanded criteria deceased	475 (5.21)	116 (6.88)	106 (4.96)	
Standard criteria deceased	6008 (65.88)	996 (59.04)	1103 (51.61)	
Living	2637 (28.91)	575 (34.08)	928 (43.43)	
Donor race:				<.001
Black	1145 (12.55)	206 (12.21)	191 (8.94)	
Others	7975 (87.45)	1481 (87.79)	1946 (91.06)	
Recipient age, years:				
Mean (SD)	44.36 (12.59)	44.69 (13.07)	46.15 (13.43)	<.001
Range	18–78	18–79	18–88	—
Recipient race:				<.001
Black	2077 (22.77)	389 (23.06)	314 (14.69)	
Others	7043 (77.23)	1298 (76.94)	1823 (85.31)	
Primary kidney disease:				
Glomerulonephritis	3417 (37.47)	632 (37.46)	776 (36.31)	0.60
Pretransplant dialysis:				<.001
1 day–1 year	1392 (15.26)	291 (17.25)	462 (21.62)	
>1 year	6515 (71.44)	1154 (68.41)	1217 (56.95)	
No dialysis	1213 (13.30)	242 (14.34)	458 (21.43)	
Pretransplant PRA:				<.001
PRA 0–20%	2408 (26.40)	446 (26.44)	981 (45.91)	
PRA > 20%	6712 (73.60)	1241 (73.56)	1156 (54.09)	
HLA mismatch				<.001
0–3	4215 (46.31)	801 (47.57)	1116 (52.30)	
More than 3	4886 (53.69)	883 (52.43)	1018 (47.70)	
Transplant year:				<.001
2003–2008	4344 (47.63)	656 (38.89)	1299 (60.79)	
2009–2013	4776 (52.37)	1031 (61.11)	838 (39.21)	
Steroids included in maintenance Immunosuppression regimen:				<.001
No	1468 (16.10)	817 (48.43)	232 (10.86)	
Yes	7652 (83.90)	870 (51.57)	1905 (89.14)	
